# Synthesis, Antigenicity Against Human Sera and Structure-Activity Relationships of Carbohydrate Moieties from *Toxocara* larvae and Their Analogues

**DOI:** 10.3390/molecules17089023

**Published:** 2012-07-30

**Authors:** Akihiko Koizumi, Kimiaki Yamano, Takashi Tsuchiya, Frank Schweizer, Fumiyuki Kiuchi, Noriyasu Hada

**Affiliations:** 1 Faculty of Pharmacy, Keio University, 1-5-30 Shibakoen, Minato-ku, Tokyo 105-8512, Japan; 2 Hokkaido Institute of Public Health, Kita-19, Nishi-12, Kita-ku, Sapporo 060-0819, Japan; 3 Departments of Chemistry and Medical Microbiology, University of Manitoba, Winnipeg, MB R3T 2N2, Canada

**Keywords:** glycoprotein, *Toxocara* larvae, host-parasite interaction, stereocontrolled synthesis, antigenicity

## Abstract

Stereocontrolled syntheses of biotin-labeled oligosaccharide portions containing the Galβ1-3GalNAc core of the TES-glycoprotein antigen obtained from larvae of the parasite *Toxocara* and their analogues have been accomplished. Trisaccharides Fuc2Meα1-2Gal4Meβ1-3GalNAcα1-OR (**A**), Fucα1-2Gal4Meβ1-3GalNAcα1-OR (**B**), Fuc2Meα1-2Galβ1-3GalNAcα1-OR (**C**), Fucα1-2Galβ1-3GalNAcα1-OR (**D**) and a disaccharide Fuc2Meα1-2Gal4Meβ1-OR (**E**) (R = biotinylated probe) were synthesized by block synthesis using 5-(methoxycarbonyl)pentyl-2,3,4,6-tetra-*O*-acetyl-β-D-galactopyranosyl-(1→3)-2-azide-4-*O*-benzyl-2-deoxy-α-D-galactopyranoside as a common glycosyl acceptor. We examined the antigenicity of these five oligosaccharides by enzyme linked immunosorbent assay (ELISA). Our results demonstrate that the *O*-methyl groups in these oligosaccharides are important for their antigenicity and the biotinylated oligosaccharides **A**, **B**, **C** and **E** have high serodiagnostic potential to detect infections caused by *Toxocara* larvae.

## 1. Introduction

In the course of our studies on unique glycoconjugates found in parasites, we have synthesized various glycosphingolipids and carbohydrate portions of glycoproteins with the aim of elucidating the mechanisms of host-parasite interactions [1,2,3,4,5,6,7]. In previous studies we have synthesized unusual carbohydrates from the parasites *Echinococcus multilocularis* [1,3,6], *Schistosoma mansoni* [2] and porcine roundworm (nematode) *Ascaris suum* [7]. In this paper we describe the synthesis of the carbohydrate portion of glycoproteins from *Toxocara canis* and *T. cati*. *T. canis* and *T. cati* are parasitic roundworms and are widely distributed in dogs and cats. Both nematodes cause severe infections in a human host affecting eyes, liver and the central nervous system [8,9].

**Figure 1:**
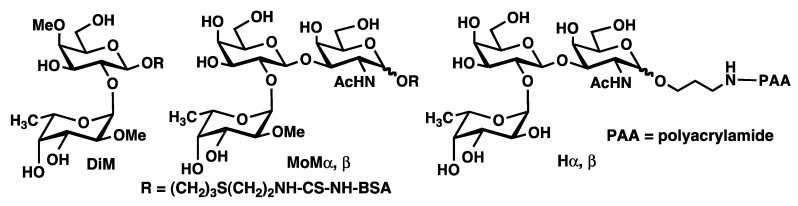
Structures of previously prepared DiM, MoM α,β and the human blood group antigen H.

Khoo *et al.*, isolated *Toxocara* excretory-secretory (TES) antigen, a family of glycoproteins that are heavily *O*-glycosylated from the culture media of *T. canis* and *T. cati* larvae [10]. The TES antigen of *T. canis* is a mixture of mucin-type glycoproteins, containing a Fuc1-2Gal1-3GalNAc structure, and it has been found that the fucose part was *O*-methylated at the 2-position and approximately 50% of the galactose part was *O*-methylated at the 4-position, *i.e.*, it contains the following two sequences: 2-*O*-Me-Fuc*p*(α1→2)-4-*O*-MeGal*p*(β1→3)-GalNAc*p* and 2-*O*-Me-Fuc*p*(α1→2)-Gal*p*(β1→3)-GalNAc*p*. Interestingly, although the di-*O*-methylated trisaccharide was found in both *T. canis* and *T. cati* the mono-*O*-methylated sugar was found only in *T. canis.* These structures are similar to the human blood group antigen H, Fuc*p*(α1→2)-Gal*p*(β1→3)-GalNAc*p* which does not have any *O*-methyl substitution. Maizels and Kosma *et al.*, synthesized di-*O*-methylated disaccharide (DiM) and mono-*O*-methylated trisaccharide (MoM α,β conjugated to BSA, Figure 1) and studied their antigenicity [11,12]. The results showed that the sera from infected patients recognized the DiM more strongly than MoM α,β [11]. However, the both groups have not studied the effects of the di-*O*-methylated trisaccharide.

**Figure 2:**
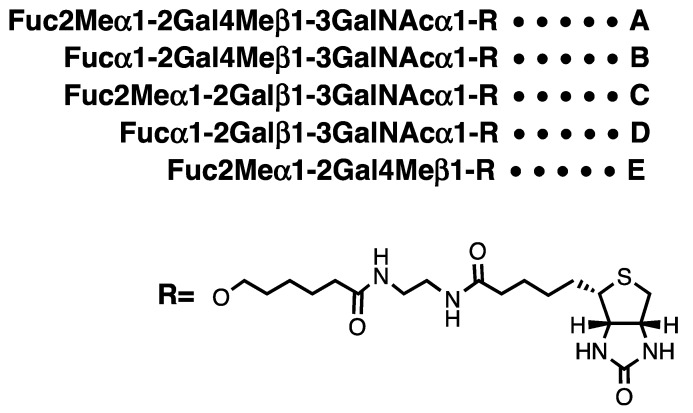
Structures of target compounds **A**–**E**.

In this paper we report the synthesis of biotin-tagged oligosaccharides **A**–**E** (Figure 2), two glycan portions of the glycoprotein antigen of *T. canis* (**A** and **C**) and three analogues (**B**, **D** and **E**), to elucidate the antigenicity of the oligosaccharides against sera of *T. canis* infected patients by enzyme-linked immunosorbent assay (ELISA).

## 2. Results and Discussion

### 2.1. Chemical Synthesis

**Scheme 1:**
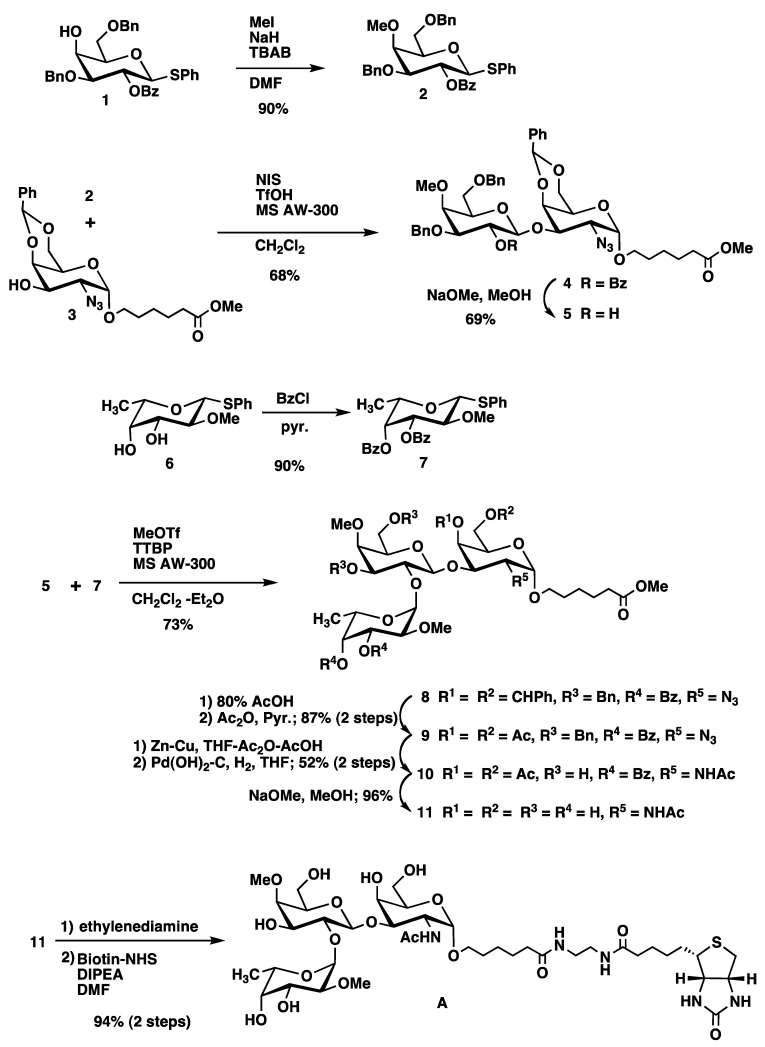
Synthesis of di-methylated trisaccharide **A**.

**Scheme 2:**
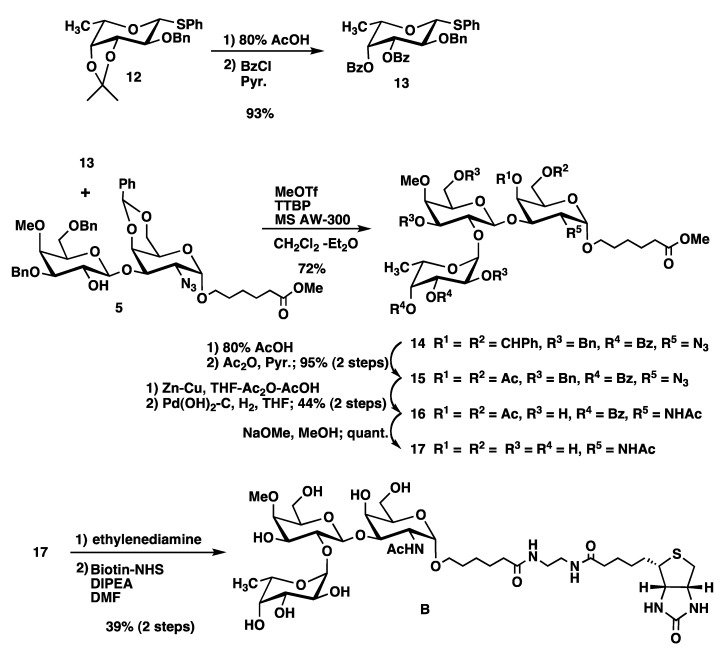
Synthesis of mono-methylated trisaccharide **B**.

**Scheme 3:**
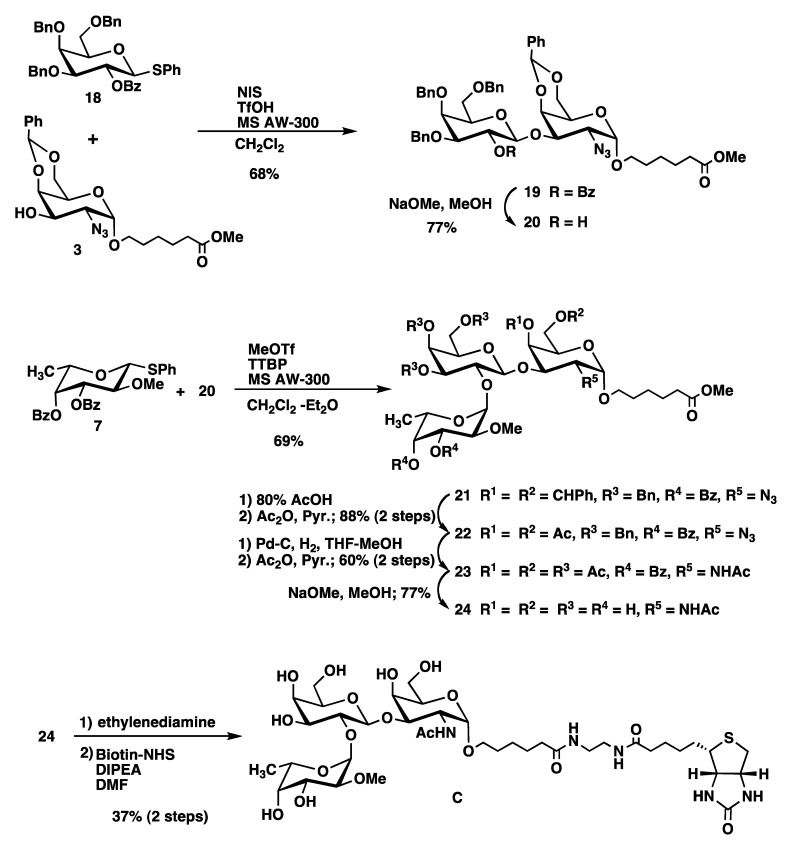
.Synthesis of mono-methylated trisaccharide **C**.

**Scheme 4:**
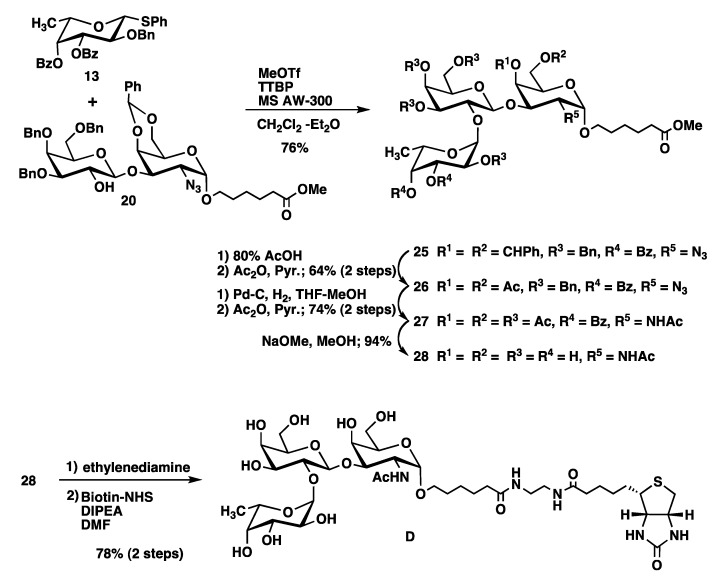
Synthesis trisaccharide **D** without *O*-methyl substitution.

**Scheme 5:**
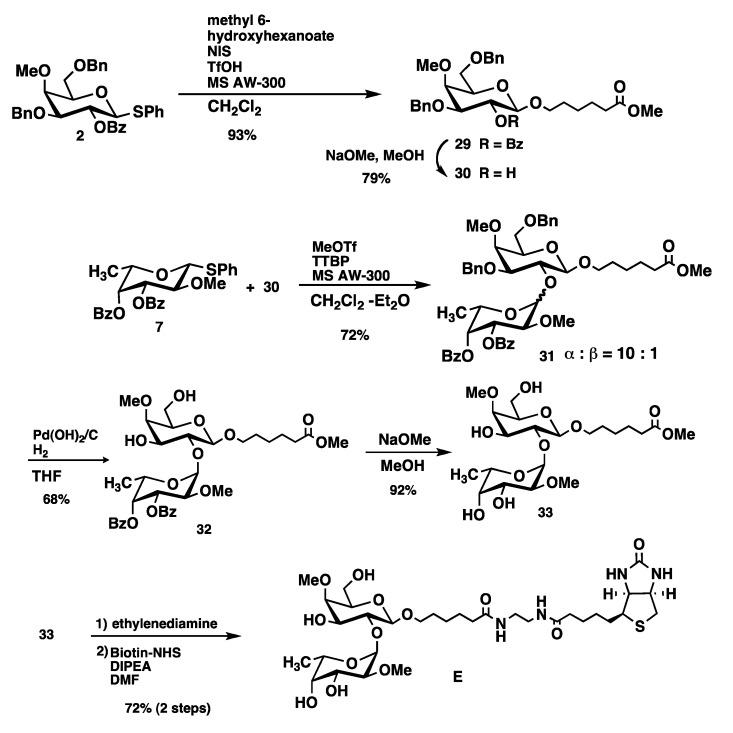
Synthesis of di-methylated disaccharide **E**.

Syntheses of the target oligosaccharides **A**–**E**: In all cases we selected 5-(methoxycarbonyl)pentyl group as the protecting group of reducing end, because this group can be conveniently used for conjugation with biotin for the use in ELISA assay as previously shown by us [1]. The synthetic routes for target compounds **A**–**E** are outlined in Scheme 1,Scheme 2,Scheme 3,Scheme 4,Scheme 5.

Monosaccharide derivative **3** was chosen as a common acceptor for the synthesis of oligosaccharides **A**–**D** (Scheme 1). Galactopyranosyl donor **2** was obtained from phenyl 3,6-di-*O*-benzyl-2-*O*-benzoyl-1-thio-β-D-galactopyranoside (**1**) [3], by methylation using methyl iodide. Glycosylation of **2** with **3** [1] in the presence of *N*-iodosuccinimide (NIS)/trifluoromethanesulfonic acid (TfOH) [13] and AW-300 molecular sieves (MS AW-300) in CH_2_Cl_2_ afforded desired disaccharide (**4**) in 68% yield. The nature of the new glycosidic linkage was determined by the vicinal coupling constant of the anomeric proton (H-1 of Gal, δ = 4.79 ppm, *J* = 8.0 Hz). Removal of the benzoyl groups in **4** under Zemplén conditions gave disaccharide acceptor **5** which was used directly for the next glycosylation step. To prepare the Fucα1-2Gal sequence α-stereoselectively, we selected 3,4-di-acyl protected fucosyl donor **7** [14], which was obtained from **6** [11] by benzoylation. Previous studies have indicated that 3,4-di-acyl-protected fucopyranosyl donors induce high α-selectivity in fucosylation [2]. Glycosylation of **5** with **7** in the presence of methyl trifluoromethanesulfonate (MeOTf) [15], tri-*tert*-butyl pyridine (TTBP) and MS AW-300 in CH_2_Cl_2_ afforded desired trisaccharide (**8**) in 73% yield. The nature of the new glycosidic linkage was determined by the coupling constant of anomeric proton (H-1 of Fuc, δ = 5.67 ppm, *J* = 3.7 Hz). Global deprotection was performed by a combination of protection/deprotection steps. At first, the benzylidene acetal of **8** was removed by acidic hydrolysis followed by *O*-acetylation using acetic anhydride in pyridine to afford **9** in 87% yield. Next, the azide group was converted to an acetamide by reduction with Zn/Cu AcOH in the presence of acetic anhydride. The obtained *N*-acylated product **9** was debenzylated by catalytic hydrogenolysis using Pearlman’s catalyst followed by *O*-acetylation to give **10** (52%), which was deacetylated to produce deprotected trisaccharide **11** in 96% yield. The 5-(methoxycarbonyl)pentyl glycoside **11** was converted into the ethylenediamine monoamide by exposure to ethylenediamine and conjugated to biotin to afford trisaccharide-biotin conjugate **A** in 94% yield (Scheme 1).

Non-methylated fucopyranosyl donor **13** was synthesized from known donor **12** [16] by two-step procedure (Scheme 2). Hydrolysis of the isopropylidene group in **12** with aqueous AcOH and subsequent benzoylation produced fucopyranosyl donor **13***.* Trisaccharide **14** was synthesized by a coupling of the fucopyranosyl donor **13** with the disaccharide acceptor **5**. The presence of an α fucosidic linkage in **14** was indicated by a doublet at δ 5.71 ppm showing small homonuclear coupling constant of 3.3 Hz in the ^1^H-NMR spectrum. Deprotection and biotinylation were performed as described for compound **A** to provide target trisaccharide **B** (Scheme 2).

The synthesis of the trisaccharide **C** is outlined in Scheme 3. As **C** does not contain the *O*-methyl substitution in the galactose residue, we chose phenyl 3,4,6-tri-*O*-benzyl-2-*O*-benzoyl-1-thio-β-D-galactopyranoside (**18**) [17] as a galactosyl donor. Glycosylation of acceptor **3** with donor **18** in the presence of NIS/TfOH provided disaccharide **19** in 68% yield. Selective removal of the benzoyl group in **19** with NaOMe produced disaccharide acceptor **20**. Fucosylation of **20** with the donor **7** afforded trisaccharide **21**, whose benzylidene acetal was removed as described for **8** and **14** to give **22** in 88% yield. Reduction of the azide group together with removal of benzyl groups of **22** was initially performed as described for compounds **9** and **15** using Zn/Cu and Pd(OH)_2_. However, this resulted only in a 24% yield of **23**. In contrast, significantly improved yield was obtained by catalytic hydrogenation of **22** with Pd-C followed by acetylation to produce the desired trisaccharide **23** in 60% yield. Deacylation and biotinylation were performed as described for compound **A** to give target trisaccharide **C** (Scheme 3).

Non-methylated trisaccharide **D** was synthesized from the glycosyl donor **13** and acceptor **20** as described for compound **C** in a good yield (Scheme 4).

Dimethylated disaccharide **E**, which does not contain a GalNAc residue was synthesized from galactosyl acceptor **30** with the fucosyl donor **7** as outlined in Scheme 5. Compound **30** was obtained by coupling of galactose-based thiophenyl glycoside **2** to methyl 6-hydroxyhexanoate and subsequent debenzoylation using standard conditions. Glycosylation of the acceptor **30** with **7** in the presence of MeOTf and TTBP afforded desired disaccharide **31** in 72% yield. Unfortunately, the anomeric ratio of the fucopyranosyl linkage was 10:1 (α:β, from ^1^H-NMR). Although we cannot properly explain the reason of it, we presume the influence by the absence of a GalNAc derivative in monosaccharide acceptor **30**. Deprotection of benzyl groups by catalytic hydrogenolysis using Pearlman’s catalyst provided **32**. It was possible to separate the major α-anomer form the minor β-glycoside at this stage. Deacylation and biotinylation were performed as described for compound **A** to provide target trisaccharide **E** in a good yield (Scheme 5).

### 2.2. Antigenicity of Oligosaccharides by ELISA

**Figure 3:**
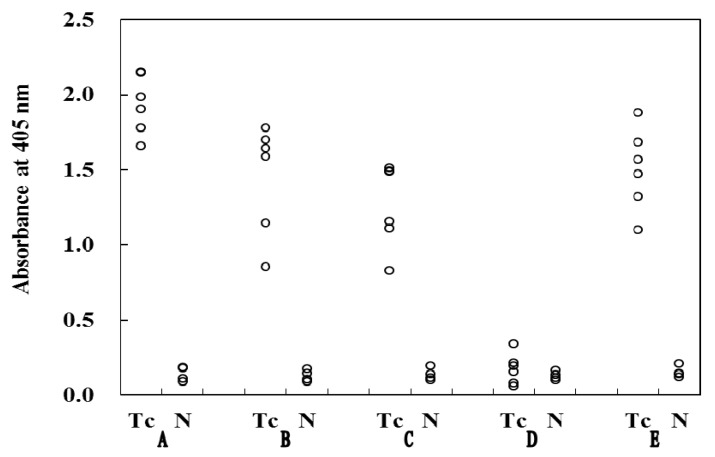
ELISA reaction between human sera and synthesized oligosaccharides **A**–**E**.Tc: toxocariasis patient group; N: normal healthy group.

The reactivity of the five biotin-labeled oligosaccharides **A** (natural), **B**, **C** (natural), **D** and **E** to patients’ sera was examined using streptavidin-coated microplates. In four of the five oligosaccharides, except for **D**, the antibody response of the *T. canis*-infected patient group (Tc) was significantly high compared with that of the normal healthy (N) group (Figure 3). However, the non-methylated trisaccharide **D** did not show any antigenicity to the patients’ sera. This is in contrast to previous findings by Kosma and Maizels, who have reported marked differences in the antigenicity between dimethylated disaccharide (DiM) and monomethylated trisaccharide (MoMα,β in the form of BSA conjugates [11]. Our study indicates that not only the natural monomethylated trisaccharide **C** containing a mono-Fuc2Me moiety but also non-natural trisaccharide **B** containing a mono-Gal4Me moiety have the antigenicity. Moreover, the antigenicity of di-methylated trisaccharide **A** was stronger than that of di-methylated disaccharide **E** indicating that the GalNAc portion contributes to the antigenicity. Significant differences of **A**–**C** and **E** were observed between Tc patient group and N group (*p* < 0.05, Student’s *t* test).

## 3. Experimental

### 3.1. General Procedures

Optical rotations were measured with a Jasco P-1020 digital polarimeter (Tokyo, Japan). ^1^H (500 MHz) and ^13^C-NMR (125 MHz) spectra were recorded with a Varian 500 FT NMR spectrometer (Palo Alto, CA, USA). Me_4_Si and acetone were used as internal standards for CDCl_3_ and D_2_O, respectively. MALDI-TOFMS was recorded on an AB SCIEX Voyager RP mass spectrometer (Framingham, MS, USA). High-resolution mass spectra were recorded on a JEOL JMS-700 (Tokyo, Japan) under FAB conditions. TLC was performed on Silica Gel 60 F254 (E. Merck, Darmstadt, Germany) with detection by quenching of UV fluorescence and by charring with 10% H_2_SO_4_. Column chromatography was carried out on Silica Gel 60. Phenyl 2-*O*-benzoyl-3,6-di-*O*-benzyl-1-thio-β-D-galactopyranoside (**1**) [3], 5-(methoxycarbonyl)pentyl 2-azide-4,6-*O*-benzylidene-2-deoxy-β-D-galactopyranoside (**3**) [1], phenyl 2-*O*-methyl-1-thio-β-L-fucopyranoside (**6**) [11], were prepared as reported.

*Phenyl 2-O-benzoyl-3*,*6-di-O-benzyl-4-O-methyl-1-thio-β-D-galactopyranoside* (**2**). Sodium hydride was added portionwise to a stirred mixture of compound **1** (253 mg, 0.45 mmol), MeI (56.5 μL, 0.91 mmol) and *n*-Bu_4_NBr (176 mg, 0.56 mmol) in DMF (5.0 mL) at −15 °C. The mixture was stirred for 3 h at −15 °C, and MeOH was added dropwise to destroy excess NaH. The mixture was diluted with EtOAc, washed with water, dried (MgSO_4_), and concentrated *in vacuo*. The product was purified by silica gel column chromatography (6:1 hexane-ethyl acetate) to give **2** (234 mg, 90%). [α]_D_ +50.6 (*c* 1.0, CHCl_3_). ^1^H-NMR (CDCl_3_): δ 8.02-7.12 (m, 20H, Ar), 5.61 (t, 1H, *J*_1,2_ = *J*_2,3_ = 9.8 Hz, H-2), 4.76 (d, 1H, *J*_1,2_ = 9.8 Hz, H-1), 4.67 and 4.53 (each d, 2H, *J*_gem_ = 12.8 Hz, PhCH_2_), 4.58 and 4.53 (each d, 2H, *J*_gem_ = 11.6 Hz, PhCH_2_), 3.79–3.67 (m, 5H, H-3, H-4, H-5, H-6a, H-6b), 3.58 (s, 1H, OCH_3_). ^13^C-NMR (CDCl_3_): δ 165.2, 137.8, 137.5, 134.0, 133.0, 129.8, 128.7, 128.5, 128.3, 127.9, 127.8, 127.6, 127.3, 87.2 (C-1), 80.8 (C-4), 77.5 (C-3), 75.2 (C-5), 73.6 (PhCH_2_), 71.6 (PhCH_2_), 70.3 (C-2), 68.5 (C-6), 61.3 (OCH_3_). MALDI-TOFMS: calcd for C_34_H_34_O_6_SNa, *m/z* 593.2; found, *m/z* 593.8 [M+Na]^+^. HR-FABMS: calcd for C_34_H_34_O_6_SNa, *m/z* 593.1974; found, *m/z* 593.1958 [M+Na]^+^.

*5-(Methoxycarbonyl)pentyl 2-O-benzoyl-3*,*6-di-O-benzyl-4-O-methyl-β-D-galacto-pyranosyl-(1→3)-2-azide-4*,*6-O-benzylidene-2-deoxy-α-D-galactopyranoside* (**4**). A mixture of **2** (203 mg, 0.37 mmol), **3** (125 mg, 0.30 mmol) and powdered MS AW300 (300 mg) in dry CH_2_Cl_2_ (3 mL) was stirred under Ar atmosphere for 2 h at room temperature, then cooled to −40 °C. NIS (160 mg, 0.71 mmol) and TfOH (6.3 μL, 71.2 μmol) were added to the mixture, which was stirred for 1 h at −40 °C, then neutralized with Et_3_N. The precipitates were filtered off and washed with CHCl_3_. The combined filtrate and washings were successively washed with saturated aqueous Na_2_S_2_O_3_ and water, dried (MgSO_4_), and concentrated. The product was purified by silica gel column chromatography (5:2 hexane-ethyl acetate) to give **4** (178 mg, 68%). [α]_D_ +94.6 (c 1.0, CHCl_3_). ^1^H-NMR (CDCl_3_): δ 8.05–7.11 (m, 20H, Ar), 5.59 (dd, 1H, *J*_1',2'_ = 8.0 Hz, *J*_2',3'_ = 9.8 Hz, H-2'), 5.45 (s, 1H, PhCH), 4.92 (d, 1H, *J*_1,2_ = 3.4 Hz, H-1), 4.79 (d, 1H, *J*_1',2'_ = 8.0 Hz H-1'), 4.68 and 4.52 (each d, 2H, *J*_gem_ = 12.2 Hz, PhCH_2_), 4.57 (s, 2H, PhCH_2_), 4.37 (d, 1H, *J*_3',4'_ = 3.1 Hz H-4), 4.15 (dd, 1H, *J*_5,6a_ = 12.4 Hz, *J*_6a,6b_ = 1.4 Hz, H-6a), 4.09 (dd, 1H, *J*_2,3_ = 11.0 Hz, *J*_3,4_ = 3.1 Hz, H-3), 3.92 (dd, 1H, *J*_5,6b_ = 12.4 Hz, *J*_6a,6b_ = 1.2 Hz, H-6b), 3.73–3.48 (m, 14H, H-2, H-5, H-3', H-4', H-5', H-6'a, H-6'b, OCH_3_×2, OCH_2_CH_2_ a), 3.47–3.42 (m, 1H, OCH_2_CH_2_ b), 2.33–2.28 (m, 2H), 1.67–1.59 (m, 4H), 1.41–1.35 (m, 2H). ^13^C-NMR (CDCl_3_: δ 174.0, 165.3, 137.9, 137.7, 137.6, 132.8, 129.8, 128.6, 128.5, 128.3, 128.2, 127.94, 127.85, 127.69, 127.65, 126.2, 102.5 (C-1'), 100.4 (CHPh) 98.7 (C-1), 79.9 (C-3'), 75.8 (C-4), 75.1 (C-4'), 74.1 (C-3), 73.69 (C-5'), 73.65 (PhCH_2_), 71.7 (PhCH_2_), 71.5 (C-2'), 69.0 (C-6), 68.6 (C-6'), 68.2 (OCH_2_CH_2_), 63.2 (C-5), 61.5 (OCH_3_), 58.8 (C-2), 33.9, 29.0, 25.6, 24.7. MALDI-TOFMS: calcd for C_48_H_55_N_3_O_13_Na, *m/z* 904.4; found, *m/z* 905.0 [M+Na]^+^. HR-FABMS: calcd for C_48_H_55_N_3_O_13_Na, *m/z* 904.3633; found, *m/z* 904.3594 [M+Na]^+^.

*5-(Methoxycarbonyl)pentyl 3*,*6-di-O-benzyl-4-O-methyl-β-D-galactopyranosyl-(1→3)-2-azide-4*,*6-O-benzylidene-2-deoxy-α-D-galactopyranoside* (**5**). A solution of **4** (301 mg, 0.34 mmol) and NaOMe (55 mg, 1.02 mmol) in MeOH (10 mL) was stirred at 50 °C for 48 h, then neutralized with Amberlite IR 120 [H^+^]. The mixture was filtered off and concentrated. The product was purified by silica gel column chromatography (2:1 hexane-ethyl acetate) to give **5** (182 mg, 69%). [α]_D_ +88.5 (c 1.0, CHCl_3_). ^1^H-NMR (CDCl_3_): δ 7.51–7.25 (m, 15H, Ar), 5.48 (s, 1H, PhCH), 5.04 (d, 1H, *J*_1,2_ = 3.5 Hz, H-1), 4.81 and 4.76 (each d, 2H, *J*_gem_ = 12.0 Hz, PhCH_2_), 4.55 (s, 2H, PhCH_2_), 4.47 (d, 1H, *J*_1',2'_ = 7.9 Hz, H-1'), 4.35 (d, 1H, *J*_3',4'_ = 3.2 Hz, H-4), 4.19 (dd, 1H, *J*_5,6a_ = 11.4 Hz, *J*_6a,6b_ = 1.2 Hz, H-6a), 4.11 (dd, 1H, *J*_2,3_ = 10.8 Hz, *J*_3,4_ = 3.2 Hz, H-3), 3.95–3.88 (m, 3H, H-2, H-2', H-6b), 3.73–3.60 (m, 9H, H-5, H-4', H-5', H-6'a, H-6'b, OCH_2_CH_2_ a, OCH_3_), 3.56 (s, 3H, OCH_3_), 3.52–3.48 (m, 1H, OCH_2_CH_2_ b), 3.45 (dd, 1H, *J*_2',3'_ = 9.8 Hz, *J*_3',4'_ = 3.0 Hz, H-3'), 2.32 (t, 2H, *J*´ = 7.7 Hz), 1.69–1.62 (m, 4H), 1.43–1.39 (m, 2H). ^13^C-NMR (CDCl_3_): δ 174.1, 138.3, 137.9, 137.7, 128.9, 128.5, 128.4, 128.1, 127.9, 127.8, 127.7, 127.6, 126.3, 105.1 (C-1'), 100.8 (CHPh) 98.5 (C-1), 81.6 (C-3'), 76.07 (C-4'), 76.06 (C-4), 75.3 (C-3), 73.8 (C-5'), 73.6 (PhCH_2_), 72.9 (PhCH_2_), 71.4 (C-2'), 69.1 (C-6), 68.6 (C-6'), 68.4 (OCH_2_CH_2_), 63.2 (C-5), 61.5 (OCH_3_), 59.1 (C-2), 51.5 (OCH_3_), 33.9, 29.1, 25.6, 24.6. MALDI-TOFMS: calcd for C_41_H_51_N_3_O_12_Na, *m/z* 800.3; found, *m/z* 800.2 [M+Na]^+^. HR-FABMS: calcd for C_41_H_51_N_3_O_12_Na, *m/z* 800.3370; found, *m/z* 800.3384 [M+Na]^+^.

*Phenyl* *3*,*4-di-O-benzoyl-2-O-methyl-1-thio-β-L-fucopyranoside* (**7**). A solution of **6** (616 mg, 2.28 mmol) and benzoyl chloride (793 μL, 6.84 mmol) in pyridine (8 mL) was stirred for 16 h at room temperature. After completion of the reaction, the mixture was poured into ice H_2_O and extracted with CHCl_3_. The extract was washed sequentially with 5% HCl, aq NaHCO_3_, and brine, dried (MgSO_4_), and concentrated. The product was purified by silica gel column chromatography (10:1 hexane-ethyl acetate) to give **7** (976 mg, 90%). [α]_D_ –63.9 (*c* 1.0, CHCl_3_). ^1^H-NMR (CDCl_3_): δ 7.96–7.26 (m, 15H, Ar), 5.63 (d, 1H, *J*_3,4_ = 2.8 Hz, H-4), 5.35 (dd, 1H, *J*_2,3_ = 9.6 Hz, *J*_3,4_ = 3.1 Hz, H-3), 4.71 (d, 1H, *J*_1,2_ = 9.5 Hz, H-1), 4.01 (q, 1H, *J*_5,6_ = 6.2 Hz, H-5), 3.63 (t, 1H, *J*_1,2_ = *J*_2,3_ = 9.5 Hz, H-2), 3.49 (s, 3H, OCH_3_), 1.32 (d, 1H, *J*_5,6_ = 6.3 Hz, H-6). ^13^C-NMR (CDCl_3_): δ 165.7, 165.5, 133.3, 133.13, 133.08, 132.6, 129.9, 129.6, 129.53, 129.49, 128.9, 128.5, 128.3, 127.8, 86.7 (C-1), 76.5 (C-2), 75.5 (C-3), 73.4 (C-5), 71.6 (C-4), 61.0 (OCH_3_), 16.7 (C-6). MALDI-TOFMS: calcd for C_27_H_26_O_6_SNa, *m/z* 501.1; found, *m/z* 501.6 [M+Na]^+^. HR-FABMS: calcd for C_27_H_27_O_6_S, *m/z* 479.1528; found, *m/z* 479.1532 [M+H]^+^.

*5-(Methoxycarbonyl)pentyl 3*,*4-di-O-benzoyl-2-O-methyl-α-L-fucopyranosyl-(1→2)-3*,*6-di-O-benzyl-4-O-methyl-β-D-galactopyranosyl-(1→3)-2-azide-4*,*6-O-benzylidene-2-deoxy-α-D-galactopyranoside* (**8**). A mixture of **5** (98.3 mg, 0.13 mmol), **7** (121 mg, 0.25 mmol) and MS AW300 (200 mg) in dry CH_2_Cl_2_-Et_2_O (1:1, 2.0 mL) was stirred for 2 h at room temperature. MeOTf (120 μL, 1.06 mmol) and TTBP (93.5 mg, 0.38 mmol) were added, and the mixture was stirred for 14 h at room temperature, then neutralized with Et_3_N. The precipitates were filtrated off and washed with CHCl_3_. The combined filtrate and washings were washed with water, dried (MgSO_4_), and concentrated. The product was purified by silica gel column chromatography (3:2 hexane-EtOAc) to give **8** (105 mg, 73%). [α]_D_ −7.9 (c 1.0, CHCl_3_). ^1^H-NMR (CDCl_3_) δ 8.02–7.23 (m, 25H, Ar), 5.67 (d, 1H, *J*_1",2"_ = 3.7 Hz, H-1"), 5.18 (d, 1H, *J*_1,2_ = 3.2 Hz, H-1), 4.74 (d, 1H, *J*_1',2'_ = 7.8 Hz, H-1'). ^13^C-NMR (CDCl_3_): δ 103.3 (C-1'), 99.2 (C-1), 97.4 (C-1"). MALDI-TOFMS: calcd for C_62_H_71_N_3_O_18_Na, *m/z* 1168.5; found, *m/z* 1168.9 [M+Na]^+^. HR-FABMS: calcd for C_62_H_71_N_3_O_18_Na, *m/z* 1168.4630; found, *m/z* 1168.4591 [M+Na]^+^.

*5-(Methoxycarbonyl)pentyl 3*,*4-di-O-benzoyl-2-O-methyl-α-L-fucopyranosyl-(1→2)-3*,*6-di-O-benzyl-4-O-methyl-β-D-galactopyranosyl-(1→3)-4*,*6-di-O-acetyl-2-azide-2-deoxy-α-D-galacto-pyranoside* (**9**). A solution of **8** (167 mg, 0.15 mmol) in 80% AcOH (5.0 mL) was stirred at 70 °C for 2 h, then diluted with toluene and concentrated. The residue was acetylated with acetic anhydride (3.0 mL) in pyridine (5.0 mL). After the reaction was quenched with MeOH, toluene was added and co-evaporated several times. The product was purified by silica gel column chromatography (3:2 hexane-ethyl acetate) to give **9** (145 mg, 87%). [α]_D_ −3.8 (c 1.0, CHCl_3_). ^1^H-NMR (CDCl_3_). δ 8.05–7.26 (m, 20H, Ar), 5.70 (d, 1H, *J*_1",2"_ = 3.6 Hz, H-1"), 5.18 (d, 1H, *J*_1,2_ = 3.4 Hz, H-1), 4.74 (d, 1H, *J*_1',2'_ = 7.5 Hz, H-1'). ^13^C-NMR (CDCl_3_): δ 103.3 (C-1'), 98.9 (C-1), 97.4 (C-1"). MALDI-TOFMS: calcd for C_59_H_71_N_3_O_20_Na, *m/z* 1164.4; found, *m/z* 1164.7 [M+Na]^+^. HR-FABMS: calcd for C_59_H_71_N_3_O_20_Na, *m/z* 1164.4529; found, *m/z* 1164.4508 [M+Na]^+^.

*5-(Methoxycarbonyl)pentyl 3*,*4-di-O-benzoyl-2-O-methyl-α-L-fucopyranosyl-(1→2)-4-O-methyl-β-D-galactopyranosyl-(1→3)-4*,*6-di-O-acetyl-2-acetamide-2-deoxy-α-D-galactopyranoside* (**10**). A mixture of **9** (145 mg, 0.13 mmol) and Zn-Cu (600 mg) in THF-AcOH-Ac_2_O (3:2:1, 6.0 mL) was stirred for 30 min. at room temperature. After completion of the reaction, the mixture was filtered and the filtrate was concentrated. The residue was purified by silica gel column chromatography (6:1 toluene-acetone) to give an acetamide compound (128 mg). A solution of this compound in THF (2.0 mL) was hydrogenolysed (0.4 MPa) under hydrogen in the presence of 10% Pd/C (100 mg) for 2 h at room temperature. The mixture was filtered and concentrated, and the product was purified by silica gel column chromatography (2:1 toluene-acetone) to give **10** (64.1 mg, 52%). [α]_D_ −11.9 (c 1.0, CHCl_3_). ^1^H-NMR (CDCl_3_). δ 7.98–7.17 (m, 10H, Ar), 5.36 (d, 1H, *J*_1",2"_ = 3.7 Hz, H-1"), 5.15 (d, 1H, *J*_1,2_ = 3.3 Hz, H-1), 4.74 (d, 1H, *J*_1',2'_ = 6.9 Hz, H-1'). ^13^C-NMR (CDCl_3_): 102.9 (C-1'), 99.1 (C-1"), 96.9 (C-1). MALDI-TOFMS: calcd for C_47_H_63_NO_21_Na, *m/z* 1000.4; found, *m/z* 1000.4 [M+Na]^+^. HR-FABMS: calcd for C_47_H_64_NO_21_, *m/z* 978.3971; found, *m/z* 978.3983 [M+H]^+^.

*5-(Methoxycarbonyl)pentyl 2-O-methyl-α-L-fucopyranosyl-(1→2)-4-O-methyl-β-D-galactopyranosyl-(1→3)-2-acetamide-2-deoxy-α-D-galactopyranoside* (**11**). A solution of **10** (64.1 mg, 65.5 μmol) and NaOMe (30 mg) in MeOH (3.0 mL) was stirred for 3 h and then neutralized with Amberlite IR 120 [H^+^]. The mixture was filtered and concentrated. The product was purified by Sephadex LH-20 column chromatography in MeOH to give **11** (43.0 mg, 96%). [α]_D_ +17.4 (c 1.0, H_2_O). ^1^H-NMR (D_2_O). δ 5.24 (d, 1H, *J*_1",2"_ = 3.8 Hz, H-1"), 4.65 (d, 1H, *J*_1,2_ = 3.4 Hz, H-1), 4.40 (d, 1H, *J*_1',2'_ = 7.7 Hz, H-1'). ^13^C-NMR (D_2_O): δ 101.6 (C-1'), 96.1 (C-1) 95.9 (C-1"). MALDI-TOFMS: calcd for C_29_H_51_NO_17_Na, *m/z* 708.3; found, *m/z* 708.8 [M+Na]^+^.

*Biotinylated trisaccharide* (**A**). Compound **11** (43.0 mg, 62.7 μmol) was dissolved in anhydrous ethylenediamine (5.0 mL) and heated at 70 °C for 44 h. The mixture was concentrated with toluene and the product was purified by Sephadex LH-20 column chromatography with H_2_O to give an amine intermediate. The amine (42.0 mg, 58.8 μmol) was dissolved in DMF (3.0 mL), and the pH was adjusted to 8–9 with DIPEA. Biotine-NHS (24.0 mg, 70.6 μmol) was added and the mixture was stirred for 13 h at room temperature. The solvent was removed by repeated co-evaporation with toluene and the product was purified by Sephadex LH-20 column chromatography with MeOH to give **A** (55.5 mg, 94%). [α]_D_ +30.8 (*c* 1.0, MeOH). ^1^H-NMR (D_2_O): δ 5.24 (d, 1H, *J*_1",2"_ = 3.9 Hz, H-1"), 4.65 (d, 1H, *J*_1,2_ = 3.5 Hz, H-1), 4.41 (d, 1H, *J*_1',2'_ = 7.7 Hz, H-1'). ^13^C-NMR (D_2_O): δ 101.6 (C-1'), 96.1 (C-1), 95.9 (C-1"). MALDI-TOFMS: calcd for C_40_H_69_N_5_O_18_SNa, *m/z* 962.4; found, *m/z* 963.0 [M+Na]^+^. HR-FABMS: calcd for C_40_H_69_N_5_O_18_SNa, *m/z* 962.4256; found, *m/z* 962.4271 [M+Na]^+^.

*Phenyl 3*,*4-di-O-benzoyl-2-O-benzyl-1-thio-β-L-fucopyranoside* (**13**). A solution of **12** (985 mg, 2.55 mmol) in 80% AcOH (20 mL) was stirred at 70 °C for 2 h, then diluted with toluene and concentrated. The residue was benzoylated with benzoyl chloride (887 μL, 7.65 mmol) in pyridine (20 mL). After the reaction was quenched with MeOH, the mixture was concentrated by repeated co-evaporation with toluene. The product was purified by silica gel column chromatography (10:1 hexane-ethyl acetate) to give **13** (1.31 g, 93%). [α]_D_ −66.2 (*c* 1.0, CHCl_3_). ^1^H-NMR (CDCl_3_): δ 7.97–7.15 (m, 20H, Ar), 5.65 (d, 1H, *J*_3,4_ = 3.0 Hz, H-4), 5.44 (dd, 1H, *J*_2,3_ = 9.4 Hz, *J*_3,4_ = 3.3 Hz, H-3), 4.81 (d, 1H, *J*_1,2_ = 9.5 Hz, H-1), 4.80 and 4.58 (each d, 2H, *J*_gem_ = 10.7 Hz, PhCH_2_), 4.04 (q, 1H, *J*_5,6_ = 6.4 Hz, H-5), 3.97 (t, 1H, *J*_1,2_ = *J*_2,3_ = 9.6 Hz, H-2), 1.33 (d, 1H, *J*_5,6_ = 6.5 Hz, H-6). ^13^C-NMR (CDCl_3_): δ 165.8, 165.5, 137.5, 133.3, 133.1, 132.90, 132.86, 130.2, 129.9, 129.6, 129.51, 129.46, 129.0, 128.5, 128.3, 128.2, 128.1, 127.81, 127.76, 87.0 (C-1), 75.3 (C-3, PhCH_2_), 74.9 (C-2), 73.4 (C-5), 71.7 (C-4), 16.8 (C-6). MALDI-TOFMS: calcd for C_33_H_30_O_6_SNa, *m/z* 577.2; found, *m/z* 577.5 [M+Na]^+^. HR-FABMS: calcd for C_33_H_31_O_6_S, *m/z* 555.1841; found, *m/z* 555.1816 [M+H]^+^.

*5-(Methoxycarbonyl)pentyl 3*,*4-di-O-benzoyl-2-O-benzyl-α-L-fucopyranosyl-(1→2)-3*,*6-di-O-benzyl-4-O-methyl-β-D-galactopyranosyl-(1→3)-2-azide-4*,*6-O-benzylidene-2-deoxy-α-D-galactopyranoside* (**14**). Compound **14** was prepared from **5** (140 mg, 0.18 mmol) and **13** (200 mg, 0.36 mmol) as described for preparation of **8**. The product was purified by silica gel column chromatography (3:1 hexane-ethyl acetate) to give **14** (157 mg, 72%). [α]_D_ −14.1 (c 1.0, CHCl_3_). ^1^H-NMR (CDCl_3_): δ 7.85–6.83 (m, 30H, Ar), 5.71 (d, 1H, *J*_1",2"_ = 3.3 Hz, H-1"), 5.19 (d, 1H, *J*_1,2_ = 3.1 Hz, H-1), 4.77 (d, 1H, *J*_1',2'_= 7.7 Hz, H-1'). ^13^C-NMR (CDCl_3_): δ 103.4 (C-1'), 99.2 (C-1), 97.1 (C-1"). MALDI-TOFMS: calcd for C_68_H_75_N_3_O_18_Na, *m/z* 1244.5; found, *m/z* 1244.6 [M+Na]^+^. HR-FABMS: calcd for C_68_H_75_N_3_O_18_Na, *m/z* 1244.4943; found, *m/z* 1244.4974 [M+Na]^+^.

*5-(Methoxycarbonyl)pentyl 3*,*4-di-O-benzoyl-2-O-benzyl-α-L-fucopyranosyl-(1→2)-3*,*6-di-O-benzyl-4-O-methyl-β-D-galactopyranosyl-(1→3)-4*,*6-di-O-acetyl-2-azide-2-deoxy-α-D-galactopyranoside* (**15**). Compound **15** was prepared from **14** (157 mg, 0.13 mmol) as described for preparation of **9**. The product was purified by silica gel column chromatography (2:1 hexane-ethyl acetate) to give **15** (148 mg, 95%). [α]_D_ −14.6 (c 1.0, CHCl_3_). ^1^H-NMR (CDCl_3_): δ 7.87–6.85 (m, 25H, Ar), 5.74 (d, 1H, *J*_1",2"_ = 3.4 Hz, H-1"), 5.06 (d, 1H, *J*_1,2_ = 3.3 Hz, H-1), 4.71 (d, 1H, *J*_1',2'_ = 7.5 Hz, H-1'). ^13^C-NMR (CDCl_3_): δ 103.4 (C-1'), 98.9 (C-1), 97.0 (C-1"). MALDI-TOFMS: calcd for C_65_H_75_N_3_O_20_Na, *m/z* 1240.4; found, *m/z* 1240.6 [M+Na]^+^. HR-FABMS: calcd for C_65_H_75_N_3_O_20_Na, *m/z* 1240.4842; found, *m/z* 1240.4852 [M+Na]^+^.

*5-(Methoxycarbonyl)pentyl 3*,*4-di-O-benzoyl-α-L-fucopyranosyl-(1→2)-4-O-methyl-β-D-galactopyranosyl-(1→3)-4*,*6-di-O-acetyl-2-acetamide-2-deoxy-α-D-galactopyranoside* (**16**). Compound **16** was prepared from **15** (148 mg, 0.12 mmol) as described for preparation of **10**. The product was purified by silica gel column chromatography (3:2 toluene-acetone) to give **16** (50.9 mg, 44%). [α]_D_ −0.7 (c 1.0, CHCl_3_). ^1^H-NMR (CDCl_3_): δ 7.99–7.16 (m, 10H, Ar), 5.26 (d, 1H, *J*_1",2"_ = 3.6 Hz, H-1"), 5.12 (d, 1H, *J*_1,2_ = 3.3 Hz, H-1), 4.44 (d, 1H, *J*_1',2'_ = 7.1 Hz, H-1'). ^13^C-NMR (CDCl_3_): δ 103.0 (C-1'), 101.2 (C-1"), 96.9 (C-1). MALDI-TOFMS: calcd for C_46_H_61_NO_21_Na, *m/z* 986.4; found, *m/z* 986.8 [M+Na]^+^.

5-(Methoxycarbonyl)pentyl α-L-fucopyranosyl-(1→2)-4-O-methyl-β-D-galacto-pyranosyl-(1→3)-2-acetamide-2-deoxy-α-D-galactopyranoside (**17**). Compound **17** was prepared from **16** (50.9 mg, 52.8 μmol) as described for preparation of **11**. The product was purified by Sephadex LH-20 column chromatography in MeOH to give **17** (35.8 mg, quant.). [α]_D_ +19.7 (c 1.0, H_2_O). ^1^H-NMR (D_2_O). δ 5.03 (d, 1H, *J*_1",2"_ = 4.2 Hz, H-1"), 4.61 (d, 1H, *J*_1,2_ = 3.5 Hz, H-1), 4.42 (d, 1H, *J*_1',2'_ = 7.6 Hz, H-1'). ^13^C-NMR (D_2_O): δ 101.5 (C-1'), 98.8 (C-1"), 96.1 (C-1). MALDI-TOFMS: calcd for C_28_H_49_NO_17_Na, *m/z* 694.3; found, *m/z* 694.7 [M+Na]^+^.

*Biotinylated trisaccharide* (**B**). Compound **B** was prepared from **17** (43.0 mg, 62.7 μmol) as described for preparation of **A**. The product was purified by Sephadex LH-20 column chromatography with MeOH to give **B** (22.7 mg, 39%). [α]_D_ +35.2 (*c* 0.5, MeOH). ^1^H-NMR (D_2_O): δ 5.03 (d, 1H, *J*_1",2"_ = 4.0 Hz, H-1"), 4.68 (d, 1H, *J*_1,2_ = 3.4 Hz, H-1), 4.42 (d, 1H, *J*_1',2'_ = 7.8 Hz, H-1'). ^13^C-NMR (D_2_O): δ 101.6 (C-1'), 96.1 (C-1), 95.9 (C-1"). MALDI-TOFMS: calcd for C_39_H_67_N_5_O_18_SNa, *m/z* 948.4; found, *m/z* 948.6 [M+Na]^+^. HR-FABMS: calcd for C_39_H_67_N_5_O_18_SNa, *m/z* 948.4100; found, *m/z* 948.4130 [M+Na]^+^.

*5-(Methoxycarbonyl)pentyl 2-O-benzoyl-3*,*4*,*6-tri-O-benzyl-α-D-galactopyranosyl-(1→3)-2-azide-4*,*6-O-benzylidene-2-deoxy-β-D-galactopyranoside* (**19**). Compound **19** was prepared from **3** (326 mg, 0.77 mmol) and **18** (600 mg, 0.93 mmol) as described for preparation of **4**. The product was purified by silica gel column chromatography (3:1 hexane-ethyl acetate) to give **19** (566 mg, 79%). [α]_D_ +88.1 (c 1.0, CHCl_3_). ^1^H-NMR (CDCl_3_): δ 8.05–7.12 (m, 25H, Ar), 5.71 (dd, 1H, *J*_1',2'_ = 8.0 Hz, *J*_2',3'_= 10.0 Hz, H-2'), 5.45 (s, 1H, PhCH), 5.01 and 4.63 (each d, 2H, *J*_gem_ = 11.8 Hz, PhCH_2_), 4.91 (d, 1H, *J*_1,2_ = 3.4 Hz, H-1), 4.82 (d, 1H, *J*_1',2'_ = 8.0 Hz H-1'), 4.64 and 4.50 (each d, 2H, *J*_gem_ = 12.4 Hz, PhCH_2_), 4.46 and 4.43 (each d, 2H, *J*_gem_ = 11.7 Hz, PhCH_2_), 4.39 (d, 1H, *J*_3',4'_ = 3.3 Hz H-4), 4.14 (dd, 1H, *J*_5,6a_ = 12.3 Hz, *J*_6a,6b_ = 1.4 Hz, H-6a), 4.09 (dd, 1H, *J*_2,3_ = 10.8 Hz, *J*_3,4_ = 3.3 Hz, H-3), 3.99 (d, 1H, *J*_3',4'_ = 2.7 Hz, H-4'), 3.89 (dd, 1H, *J*_5,6b_ = 12.3 Hz, *J*_6a,6b_ = 1.4 Hz, H-6b), 3.72–3.63 (m, 9H, H-2, H-5, H-3', H-6'a, H-6'b, OCH_3_, OCH_2_CH_2_ a), 3.57 (s, 1H, H-5'), 3.45–3.41 (m, 1H, OCH_2_CH_2_ b), 2.30 (t, 2H, *J*´ = 7.3 Hz), 1.66–1.57 (m, 4H), 1.40–1.35 (m, 2H). ^13^C-NMR (CDCl_3_): δ 174.1, 165.3, 138.5, 137.9, 137.7, 137.6, 132.8, 130.3, 129.8, 128.6, 128.5, 128.3, 128.22, 128.20, 127.97, 127.92, 127.85, 127.70, 127.67, 127.65, 126.2, 102.6 (C-1'), 100.5 (CHPh), 98.7 (C-1), 80.1 (C-3'), 75.8 (C-4), 74.5 (C-3), 74.4 (PhCH_2_), 73.9 (C-5), 73.6 (PhCH_2_), 72.7 (C-4'), 71.9 (PhCH_2_), 71.7 (C-2'), 69.12 (C-6'), 69.06 (C-6), 68.2 (OCH_2_CH_2_), 63.2 (C-5'), 58.6 (C-2), 51.5 (OCH_3_), 33.9, 29.0, 25.6, 24.7. MALDI-TOFMS: calcd for C_54_H_59_N_3_O_13_Na, *m/z* 980.4; found, *m/z* 981.4 [M+Na]^+^. HR-FABMS: calcd for C_54_H_59_N_3_O_13_Na, *m/z* 980.3946; found, *m/z* 980.3924 [M+Na]^+^.

*5-(Methoxycarbonyl)pentyl 3*,*4*,*6-tri-O-benzyl-α-D-galactopyranosyl-(1→3)-2-azide-4*,*6-O-benzylidene-2-deoxy-β-D-galactopyranoside* (**20**). Compound **20** was prepared from **19** (200 mg, 0.22 mmol) as described for preparation of **5**. The product was purified by silica gel column chromatography (5:2 hexane–ethyl acetate) to give **20** (87.3 mg, 41%). [α]_D_ +61.0 (*c* 1.0, CHCl_3_). ^1^H-NMR (CDCl_3_): δ 7.51–7.24 (m, 20H, Ar), 5.47 (s, 1H, PhCH), 5.02 (d, 1H, *J*_1,2_ = 3.6 Hz, H-1), 4.94 and 4.60 (each d, 2H, *J*_gem_ = 11.6 Hz, PhCH_2_), 4.81 and 4.74 (each d, 2H, *J*_gem_ = 11.9 Hz, PhCH_2_), 4.49 (d, 1H, *J*_1',2'_ = 7.7 Hz, H-1'), 4.45 and 4.41 (each d, 2H, *J*_gem_ = 11.9 Hz, PhCH_2_), 4.38 (d, 1H, *J*_3',4'_ = 3.2 Hz, H-4), 4.18 (dd, 1H, *J*_5,6a_ = 12.4 Hz, *J*_6a,6b_ = 1.4 Hz, H-6a), 4.12 (dd, 1H, *J*_2,3_ = 10.8 Hz, *J*_3,4_ = 3.3 Hz, H-3), 4.05 (dd, 1H, *J*_1',2'_ = 8.3 Hz , *J*_2',3'_ = 9.1 Hz, H-2'), 3.95–3.88 (m, 3H, H-2, H-6b, H-4'), 3.72–3.68 (m, 1H, OCH_2_CH_2_ a), 3.66–3.55 (m, 7H, H-5, H-5', H-6'a, H-6'b, OCH_3_), 3.51–3.47 (m, 2H, H-3', OCH_2_CH_2_ b), 2.32 (t, 2H, *J*´ = 7.4 Hz), 1.69–1.61 (m, 4H), 1.43–1.38 (m, 2H). ^13^C-NMR (CDCl_3_): δ 174.1, 138.6, 138.4, 137.9, 137.7, 128.8, 128.5, 128.4, 128.3, 128.2, 128.1, 127.9, 127.8, 127.7, 127.6, 126.3, 105.2 (C-1'), 100.8 (CHPh) 98.5 (C-1), 81.8 (C-3'), 76.0 (C-4), 75.7 (C-3), 74.6 (PhCH_2_), 73.9 (C-5'), 73.7 (C-4'), 73.5 (PhCH_2_), 73.0 (PhCH_2_), 71.5 (C-2'), 69.2 (C-6'), 69.1 (C-6), 68.3 (OCH_2_CH_2_), 63.1 (C-5), 58.9 (C-2), 51.5 (OCH_3_), 33.9, 29.1, 25.6, 24.6. MALDI-TOFMS: calcd for C_47_H_55_N_3_O_12_Na, *m/z* 876.4; found, *m/z* 876.5 [M+Na]^+^. HR-FABMS: calcd for C_47_H_55_N_3_O_12_Na, *m/z* 876.3683; found, *m/z* 876.3721 [M+Na]^+^.

*5-(Methoxycarbonyl)pentyl 3*,*4-di-O-benzoyl-2-O-methyl-α-L-fucopyranosyl-(1→2)-3*,*4*,*6-tri-O-benzyl-β-D-galactopyranosyl-(1→3)-2-azide-4*,*6-O-benzylidene-2-deoxy-α-D-galactopyranoside* (**21**). Compound **21** was prepared from **20** (119 mg, 0.14 mmol) and **7** (133 mg, 0.28 mmol) as described for preparation of **8**. The product was purified by silica gel column chromatography (25:1 toluene-acetone) to give **21** (118 mg, 69%). [α]_D_ +1.1 (c 1.0, CHCl_3_). ^1^H-NMR (CDCl_3_): δ 8.03–7.22 (m, 30H, Ar), 5.72 (d, 1H, *J*_1",2"_ = 3.5 Hz, H-1"), 5.17 (d, 1H, *J*_1,2_ = 3.1 Hz, H-1), 4.76 (d, 1H, *J*_1',2'_ = 7.5 Hz, H-1'). ^13^C-NMR (CDCl_3_): δ 103.4 (C-1'), 99.3 (C-1), 97.5 (C-1"). MALDI-TOFMS: calcd for C_68_H_75_N_3_O_18_Na, *m/z* 1244.5; found, *m/z* 1244.1 [M+Na]^+^. HR-FABMS: calcd for C_68_H_75_N_3_O_18_Na, *m/z* 1244.4943; found, *m/z* 1244.4974 [M+Na]^+^.

*5-(Methoxycarbonyl)pentyl 3*,*4-di-O-benzoyl-2-O-methyl-α-L-fucopyranosyl-(1→2)-3*,*4*,*6-tri-O-benzyl-β-D-galactopyranosyl-(1→3)-4*,*6-di-O-acetyl-2-azide-2-deoxy-α-D-galactopyranoside* (**22**). Compound **22** was prepared from **21** (130 mg, 106 μmol) as described for preparation of **9**. The product was purified by silica gel column chromatography (2:1 hexane-ethyl acetate) to give **22** (114 mg, 88.4%). [α]_D_ −1.1 (c 1.0, CHCl_3_). ^1^H-NMR (CDCl_3_): δ 8.05–7.26 (m, 25H, Ar), 5.73 (d, 1H, *J*_1",2"_ = 3.8 Hz, H-1"), 5.05 (d, 1H, *J*_1,2_ = 3.5 Hz, H-1), 4.75 (d, 1H, *J*_1',2'_ = 7.7 Hz, H-1'). ^13^C-NMR (CDCl_3_): δ 103.1 (C-1'), 98.9 (C-1), 97.4 (C-1"). MALDI-TOFMS: calcd for C_65_H_75_N_3_O_20_Na, *m/z* 1240.5; found, *m/z* 1240.6 [M+Na]^+^. HR-FABMS: calcd for C_65_H_75_N_3_O_20_Na, *m/z* 1240.4842; found, *m/z* 1240.4852 [M+Na]^+^.

*5-(Methoxycarbonyl)pentyl 3*,*4-di-O-benzoyl-2-O-methyl-α-L-fucopyranosyl-(1→2)-3*,*4*,*6-tri-O-acetyll-β-D-galactopyranosyl-(1→3)-4*,*6-di-O-acetyl-2-acetamide-2-deoxy-α-D-galactopyranoside* (**23**). A solution of **22** (98.1 mg, 80.5 μmol) in MeOH–THF (1:1, 3 mL) was hydrogenolysed (0.4 MPa) under hydrogen in the presence of 10% Pd/C (150 mg) for 18 h at room temperature, then filtered and concentrated. The residue was acetylated with acetic anhydride (2 mL) in pyridine (3 mL). The reaction mixture was poured into ice H_2_O and extracted with CHCl_3_. The extract was washed sequentially with 5% HCl, aq NaHCO_3_, and brine, dried (MgSO_4_), and concentrated. The residue was purified by silica gel column chromatography (4:1 toluene-acetone) to give **23** (61.3 mg, 60%). [α]_D_ −10.4 (c 0.4, CHCl_3_). ^1^H-NMR (CDCl_3_): δ 7.97–7.18 (m, 10H, Ar), 5.35 (d, 1H, *J*_1",2"_ = 3.5 Hz, H-1"), 5.15 (d, 1H, *J*_1,2_ = 3.3 Hz, H-1), 4.51 (d, 1H, *J*_1',2'_ = 7.5 Hz, H-1'). ^13^C-NMR (CDCl_3_): δ 102.8 (C-1'), 99.1 (C-1"), 96.9 (C-1). MALDI-TOFMS: calcd for C_46_H_61_NO_21_Na, *m/z* 986.4; found, *m/z* 987.2 [M+Na]^+^. HR-FABMS: calcd for C_46_H_61_NO_21_Na, *m/z* 986.3634; found, *m/z* 986.3618 [M+Na]^+^.

*5-(Methoxycarbonyl)pentyl 2-O-methyl-α-L-fucopyranosyl-(1→2)-β-D-galactopyranosyl-(1→3)-2-acetamide-2-deoxy-α-D-galactopyranoside* (**24**). Compound **24** was prepared from **23** (18.7 mg, 19.4 μmol) as described for preparation of **11**. The product was purified by Sephadex LH-20 column chromatography with MeOH to give **24** (10.0 mg, 77%). [α]_D_ +24.7 (c 0.2, H_2_O). ^1^H-NMR (D_2_O): δ 5.27 (d, 1H, *J*_1",2"_ = 4.0 Hz, H-1"), 4.66 (d, 1H, *J*_1,2_ = 3.4 Hz, H-1), 4.44 (d, 1H, *J*_1',2'_ = 7.7 Hz, H-1'). ^13^C-NMR (D_2_O): δ 101.7 (C-1'), 96.1 (C-1), 95.9 (C-1"). MALDI-TOFMS: calcd for C_28_H_49_NO_17_Na, *m/z* 694.3; found, *m/z* 694.9 [M+Na]^+^.

*Biotinylated trisaccharide* (**C**). Compound **C** was prepared from **24** (10.0 mg, 14.9 μmol) as described for preparation of **A**. The product was purified by Sephadex LH-20 column chromatography with MeOH to give **C** (6.1 mg, 37%). [α]_D_ +8.9 (*c* 0.2, MeOH).^1^H-NMR (D_2_O): δ 5.27 (d, 1H, *J*_1",2"_ = 3.9 Hz, H-1"), 4.66 (d, 1H, *J*_1,2_ = 3.2 Hz, H-1), 4.44 (d, 1H, *J*_1',2'_ = 7.4 Hz, H-1'). ^13^C-NMR (D_2_O): δ 101.6 (C-1'), 96.1 (C-1), 95.9 (C-1"). MALDI-TOFMS: calcd for C_39_H_67_N_5_O_18_SNa, *m/z* 948.4; found, *m/z* 948.8 [M+Na]^+^. HR-FABMS: calcd for C_39_H_67_N_5_O_18_SNa, *m/z* 948.4100; found, *m/z* 948.4132 [M+Na]^+^.

*5-(Methoxycarbonyl)pentyl 3*,*4-di-O-benzoyl-2-O-benzyl-α-L-fucopyranosyl-(1→2)-3*,*4*,*6-tri-O-benzyl-β-D-galactopyranosyl-(1→3)-2-azide-4*,*6-O-benzylidene-2-deoxy-α-D-galactopyranoside* (**25**). Compound **25** was prepared from **20** (152 mg, 0.18 mmol) and **13** (197 mg, 0.356 mmol) as described for preparation of **8**. The product was purified by silica gel column chromatography (2:1 hexane-ethyl acetate) to give **25** (176 mg, 76%). ^1^H-NMR (CDCl_3_): δ 7.86–6.82 (m, 35H, Ar), 5.76 (d, 1H, *J*_1",2"_ = 3.5 Hz, H-1"), 5.18 (d, 1H, *J*_1,2_ = 3.3 Hz, H-1), 4.79 (d, 1H, *J*_1',2'_ = 7.8 Hz, H-1'). ^13^C-NMR (CDCl_3_): δ 103.4 (C-1'), 99.3 (C-1), 97.2 (C-1"). MALDI-TOFMS: calcd for C_74_H_79_N_3_O_18_Na, *m/z* 1320.5; found, *m/z* 1321.6 [M+Na]^+^. HR-FABMS: calcd for C_74_H_80_N_3_O_18_, *m/z* 1298.5437; found, *m/z* 1298.5442 [M+H]^+^.

*5-(Methoxycarbonyl)pentyl 3*,*4-di-O-benzoyl-2-O-benzyl-α-L-fucopyranosyl-(1→2)-3*,*4*,*6-tri-O-benzyl-β-D-galactopyranosyl-(1→3)-4*,*6-di-O-acetyl-2-azide-2-deoxy-α-D-galactopyranoside* (**26**). Compound **26** was prepared from **25** (176 mg, 0.14 mmol) as described for preparation of **9**. The product was purified by silica gel column chromatography (2:1 hexane–ethyl acetate) to give **26** (113 mg, 64%). [α]_D_ −11.2 (c 1.0, CHCl_3_). ^1^H-NMR (CDCl_3_): δ 7.87–6.83 (m, 30H, Ar), 5.77 (d, 1H, *J*_1",2"_ = 3.4 Hz, H-1"), 5.06 (d, 1H, *J*_1,2_ = 3.5 Hz, H-1), 4.77 (d, 1H, *J*_1',2'_ = 7.4 Hz, H-1'). ^13^C-NMR (CDCl_3_): δ 103.2 (C-1'), 98.9 (C-1), 97.1 (C-1"). MALDI-TOFMS: calcd for C_71_H_79_N_3_O_20_Na, *m/z* 1316.5; found, *m/z* 1316.9 [M+Na]^+^. HR-FABMS: calcd for C_71_H_79_N_3_O_20_Na, *m/z* 1316.5155; found, *m/z* 1316.5186 [M+Na]^+^.

*5-(Methoxycarbonyl)pentyl 3*,*4-di-O-benzoyl-α-L-fucopyranosyl-(1→2)-3*,*4*,*6-tri-O-acetyl-β-D-galactopyranosyl-(1→3)-4*,*6-O-acetyl-2-acetamide-2-deoxy-α-D-galactopyranoside* (**27**). Compound **27** was prepared from **26** (176 mg, 135 μmol) as described for preparation of **23**. The product was purified by silica gel column chromatography (5:1 toluene–acetone) to give **27** (110 mg, 74%). [α]_D_ −5.1 (c 0.5, CHCl_3_). ^1^H-NMR (CDCl_3_): δ 7.97–7.18 (m, 10H, Ar), 5.35 (br. s, 1H, H-1"), 5.06 (d, 1H, *J*_1,2_ = 3.2 Hz, H-1), 4.46 (d, 1H, *J*_1',2'_ = 7.1 Hz, H-1'). ^13^C-NMR (CDCl_3_): δ 102.9 (C-1'), 101.0 (C-1"), 97.0 (C-1). MALDI-TOFMS: calcd for C_45_H_59_NO_21_Na, *m/z* 972.3; found, *m/z* 972.7 [M+Na]^+^.

*5-(Methoxycarbonyl)pentyl* α*-L-fucopyranosyl-(1→2)-β-D-galactopyranosyl-(1→3)-2-acetamide-2-deoxy-α-D-galactopyranoside* (**28**). Compound **28** was prepared from **27** (25.8 mg, 27.2 μmol) as described for preparation of **11**. The product was purified by Sephadex LH-20 column chromatography in MeOH to give **28** (16.8 mg, 94%). [α]_D_ +22.3 (*c* 0.4, H_2_O). ^1^H-NMR (D_2_O): δ 5.05 (d, 1H, *J*_1",2"_ = 4.0 Hz, H-1"), 4.68 (d, 1H, *J*_1,2_ = 3.4 Hz, H-1), 4.45 (d, 1H, *J*_1',2'_ = 7.7 Hz, H-1'). ^13^C-NMR (D_2_O): δ 101.6 (C-1'), 98.8 (C-1"), 96.1 (C-1). MALDI-TOFMS: calcd for C_27_H_47_NO_17_Na, *m/z* 680.3; found, *m/z* 681.2 [M+Na]^+^.

*Biotinylated trisaccharide* (**D**). Compound **D** was prepared from **28** (18.6 mg, 25.5 μmol) as described for preparation of **A**. The product was purified by Sephadex LH-20 column chromatography with MeOH to give **D** (18.2 mg, 78%). [α]_D_ +26.5 (*c* 0.5, MeOH). ^1^H-NMR (D_2_O): δ 5.05 (d, 1H, *J*_1",2"_ = 4.0 Hz, H-1"), 4.69 (d, 1H, *J*_1,2_ = 3.1 Hz, H-1), 4.46 (d, 1H, *J*_1',2'_ = 7.7 Hz, H-1'). ^13^C-NMR (D_2_O): δ 101.6 (C-1'), 98.8 (C-1"), 96.1 (C-1). MALDI-TOFMS: calcd for C_38_H_65_N_5_O_18_SNa, *m/z* 934.4; found, *m/z* 934.5 [M+Na]^+^. HR-FABMS: calcd for C_38_H_65_N_5_O_18_SNa, *m/z* 934.3943; found, *m/z* 934.3956 [M+Na]^+^.

*5-(Methoxycarbonyl)pentyl 2-O-benzoyl-3*,*6-di-O-benzyl-4-O-methyl-β-D-galactopyranoside* (**29**). Compound **29** was prepared from methyl 6-hydroxyhexanoate (134 mg, 0.918 mmol) and **2** (575 mg, 1.01 mmol) as described for preparation of **4**. The product was purified by silica gel column chromatography (4:1 hexane–ethyl acetate) to give **29** (520 mg, 93%). [α]_D_ +7.2 (*c* 1.0, CHCl_3_). ^1^H-NMR (CDCl_3_): δ 8.02–7.12 (m, 15H, Ar), 5.52 (dd, 1H, *J*_1,2_ = 7.8 Hz, *J*_2,3_ = 10.2 Hz, H-2), 4.69 and 4.53 (each d, 2H, *J*_gem_ = 12.4 Hz, PhCH_2_), 4.60 and 4.56 (each d, 2H, *J*_gem_ = 11.7 Hz, PhCH_2_), 4.44 (d, 1H, *J*_1,2_ = 7.8 Hz, H-1), 3.79–3.67 (m, 3H, H-4, H-6a, OCH_2_CH_2_ a), 3.67 (dd, 1H, *J*_5,6b_ = 9.1 Hz, *J*_6a,6b_ = 5.5 Hz, H-6b), 3.63–3.59 (m, 8H, H-3, H-5, OCH_3_×2), 3.41–3.37 (m, 1H, OCH_2_CH_2_ b), 2.08–1.95 (m, 2H), 1.51–1.39 (m, 4H), 1.20–1.14 (m, 2H). ^13^C-NMR (CDCl_3_): δ 174.1, 165.2, 137.8, 137.7, 132.9, 130.3, 129.8, 128.5, 128.29, 128.28, 128.0, 127.9, 127.7, 101.4 (C-1), 79.6 (C-3), 74.9 (C-4), 73.7 (PhCH_2_), 73.4 (C-5), 71.8 (C-2), 71.5 (PhCH_2_), 69.0 (OCH_2_CH_2_), 68.3 (C-6), 61.3 (OCH_3_), 51.3 (OCH_3_), 33.7, 29.0, 25.3, 24.4. MALDI-TOFMS: calcd for C_35_H_42_O_9_Na, *m/z* 629.3; found, *m/z* 629.7 [M+Na]^+^. HR-FABMS: calcd for C_35_H_42_O_9_Na, *m/z* 629.2727; found, *m/z* 629.2690 [M+Na]^+^.

*5-(Methoxycarbonyl)pentyl 3*,*6-di-O-benzyl-4-O-methyl-β-D-galactopyranoside* (**30**). Compound **30** was prepared from **29** (199 mg, 0.328 mmol) as described for preparation of **5**. The product was purified by silica gel column chromatography (4:1 hexane-ethyl acetate) to give **30** (130 mg, 79%). [α]_D_ −5.5 (*c* 1.0, CHCl_3_). ^1^H-NMR (CDCl_3_): δ 40–7.27 (m, 10H, Ar), 4.78 and 4.72 (each d, 2H, *J*_gem_ = 12.0 Hz, PhCH_2_), 4.57 and 4.53 (each d, 2H, *J*_gem_ = 11.7 Hz, PhCH_2_), 4.20 (d, 1H, *J*_1,2_ = 7.6 Hz, H-1), 3.89-3.81 (m, 2H, H-2, OCH_2_CH_2_ a), 3.72 (dd, 1H, *J*_5,6a_ = 9.1 Hz, *J*_6a,6b_ = 7.6 Hz, H-6a), 3.67–3.61 (m, 5H, H-4, H-6b, OCH_3_), 3.57–3.55 (m, 4H, H-5, OCH_3_), 3.50–3.45 (m, 1H, OCH_2_CH_2_ b), 3.40 (dd, 1H, *J*_2,3_ = 9.8 Hz, *J*_3,4_ = 2.9 Hz, H-3), 2.30 (t, 2H, *J*´ = 7.5 Hz), 1.66–1.59 (m, 4H), 1.40–1.35 (m, 2H). ^13^C-NMR (CDCl_3_): δ 174.2, 138.2, 137.9, 128.50, 128.47, 127.89, 127.85, 127.8, 127.7, 103.1 (C-1), 81.7 (C-3), 75.4 (C-4), 73.6 (PhCH_2_), 73.4 (C-5), 72.4 (PhCH_2_), 71.3 (C-2), 69.4 (OCH_2_CH_2_), 68.3 (C-6), 61.2 (OCH_3_), 51.5 (OCH_3_), 33.9, 29.1, 25.5, 24.6. MALDI-TOFMS: calcd for C_28_H_38_O_8_Na, *m/z* 525.3; found, *m/z* 526.1 [M+Na]^+^. HR-FABMS: calcd for C_28_H_39_O_8_, *m/z* 503.2645; found, *m/z* 503.2636 [M+H]^+^.

*5-(Methoxycarbonyl)pentyl 3*,*4-di-O-benzoyl-2-O-methyl-L-fucopyranosyl-(1→2)-3*,*6-di-O-benzyl-4-O-methyl-β-D-galactopyranoside* (**31**). Compound **31** was prepared from **30** (130 mg, 0.26 mmol) and **7** (248 mg, 0.52 mmol) as described for preparation of **8**. The product was purified by silica gel column chromatography (30:1 toluene-acetone) to give **31** (105 mg, 72%). MALDI-TOFMS: calcd for C_49_H_58_O_14_Na, *m/z* 893.4; found, *m/z* 893.8 [M+Na]^+^. HR-FABMS: calcd for C_49_H_58_O_14_Na, *m/z* 893.3724; found, *m/z* 893.3768 [M+Na]^+^.

*5-(Methoxycarbonyl)pentyl 3*,*4-di-O-benzoyl-2-O-methyl-α-L-fucopyranosyl-(1→2)-4-O-methyl-β-D-galactopyranoside* (**32**). A solution of **31** (113 mg, 87.3 μmol) in THF (2.0 mL) was hydrogenolysed (0.4 MPa) under hydrogen in the presence of 10% Pd/C (150 mg) for 2 h at room temperature. The mixture was filtered and concentrated, and the product was purified by silica gel column chromatography (4:1 toluene–acetone) to give **32** (88.2 mg, 68%). [α]_D_ −115 (*c* 1.0, CHCl_3_). ^1^H-NMR (CDCl_3_): δ 8.06–7.31 (m, 10H, Ar), 5.69 (dd, 1H, *J*_2',3'_ = 10.1 Hz, *J*_3',4'_ = 3.2 Hz, H-3'), 5.65 (d, 1H, *J*_3',4'_ = 3.3 Hz, H-4'), 5.41 (d, 1H, *J*_1',2'_ = 3.6 Hz, H-1'), 4.56 (q, 1H, *J*_5',6'_ = 6.3 Hz, H-5'), 4.36 (d, 1H, *J*_1,2_ = 7.5 Hz, H-1), 3.97–3.88 (m, 3H, H-6a, H-2', OCH_2_CH_2_ a), 3.81–3.71 (m, 3H, H-2, H-3, H-6b), 3.66 (s, 3H, OCH_3_), 3.59 (s, 3H, OCH_3_), 3.58–3.55 (m, 3H, H-4, H-5, OCH_2_CH_2_ b), 3.53 (s, 3H, OCH_3_), 2.33 (t, 2H, *J*´ = 7.5 Hz), 1.70–1.63 (m, 4H), 1.45–1.39 (m, 2H), 1.20 (d, 3H, *J*_5',6'_= 6.6 Hz, H-6'). ^13^C-NMR (CDCl_3_): δ 174.2, 165.9, 165.6, 133.3, 133.1, 129.8, 129.72, 129.68, 129.6, 128.5, 128.3, 101.9 (C-1), 99.7 (C-1'), 81.1 (C-3), 78.6 (C-4), 77.4 (C-2'), 75.23 (C-5), 75.16 (C-2), 72.2 (C-4'), 71.5 (C-3'), 69.6 (C-6), 65.4 (C-5'), 62.3 (OCH_2_CH_2_), 61.6 (OCH_3_), 60.2 (OCH_3_), 51.5 (OCH_3_), 33.9, 29.5, 25.6, 24.7, 16.1 (C-6'). MALDI-TOFMS: calcd for C_35_H_46_O_14_Na, *m/z* 713.3; found, *m/z* 713.9 [M+Na]^+^. HR-FABMS: calcd for C_35_H_46_O_14_Na, *m/z* 713.2785; found, *m/z* 713.2770 [M+Na]^+^.

*5-(Methoxycarbonyl)pentyl 2-O-methyl-α-L-fucopyranosyl-(1→2)-4-O-methyl-β-D-galactopyranoside* (**33**). Compound **33** was prepared from **32** (88.2 mg, 0.13 mmol) as described for preparation of **11**. The product was purified by Sephadex LH-20 column chromatography with MeOH to give **33** (56.9 mg, 92%). [α]_D_ −78.6 (*c* 1.0, MeOH). ^1^H-NMR (CDCl_3_): δ 5.36 (d, 1H, *J*_1',2'_ = 3.4 Hz, H-1'), 4.29 (d, 1H, *J*_1',2'_ = 7.7 Hz, H-1'). ^13^C-NMR (CDCl_3_): δ 102.0 (C-1), 97.7 (C-1'). MALDI-TOFMS: calcd for C_21_H_38_O_12_Na, *m/z* 505.2; found, *m/z* 505.9 [M+Na]^+^. HR-FABMS: calcd for C_21_H_38_O_12_Na, *m/z* 505.2261; found, *m/z* 505.2242 [M+Na]^+^.

*Biotinylated trisaccharide* (**E**). Compound **E** was prepared from **33** (56.9 mg, 118 mmol) as described for preparation of **A**. The product was purified by Sephadex LH-20 column chromatography with H_2_O to give **E** (51.2 mg, 72%). [α]_D_ –30.9 (*c* 1.0, MeOH). ^1^H-NMR (D_2_O): δ 5.29 (d, 1H, *J*_1',2'_ = 3.8 Hz, H-1'), 4.25 (d, 1H, *J*_1',2'_ = 7.9 Hz, H-1). ^13^C-NMR (D_2_O): δ 102.5 (C-1), 97.3 (C-1'). MALDI-TOFMS: calcd for C_32_H_56_N_4_O_13_SNa, *m/z* 759.3; found, *m/z* 760.1 [M+Na]^+^. HR-FABMS: calcd for C_32_H_56_N_4_O_13_SNa, *m/z* 759.3462; found, *m/z* 759.3503 [M+Na]^+^.

### 3.2. Serum Samples

Serum samples examined by ELISA were obtained from 6 patients who were confirmed to have *Toxocara canis*-visceral larva migrans (Tc) and 4 normal healthy individuals (NH).

### 3.3. ELISA Protocol

ELISA was performed as previously described [1,18]. Biotin-labeled oligosaccharides A-E in H_2_O (13 pmol per well) were added to the wells of flat-bottomed microplates (Streptavidin C96, No. 236001; Nunc, Roskilde, Denmark) coated with streptavidin, and these plates were incubated for 1 h at 37 °C. After the coating solution was discarded, the microplates were washed with 0.05% Tween-PBS (250 μL per well). Serum samples diluted 1:250 with 0.05% Tween-PBS (200 μL per well) were then added to the wells of the microplates and incubated overnight at 4 °C. After being washed with 0.05% Tween-PBS, 200 μL of anti-human IgG/HRP (P0214; DakoCytomation, Glostrup, Denmark; 1:1,000 in 0.05% Tween-PBS) was added, and the microplates were incubated for 1 h at 37 °C. After further washing, bound antibodies were detected by the addition of 2,2'-azino-di(3-ethyl-benzthiazoline-6-sulfonate (ABTS) peroxidase substrate solution (KPL, Gaithersburg, MD, USA, 200 μL per well). After incubation period of 8 min at 37 °C, the reaction was stopped by the addition of 1% sodium dodecyl sulphate (SDS), and the absorbance (A) values were read at 405 nm on a microplate reader (Model 680; BioRad, Hercules, CA, USA).

## 4. Conclusions

We have prepared oligosaccharide-biotin conjugates **A**–**E** in order to study the antigenicity of putative carbohydrate sequences at the parasite *T. canis* and their analogues. Antigenicity of these compounds was examined by ELISA. Mono- or di-*O*-methylated forms showed good serodiagnostic potential to detect infections caused by *T. canis*. These results demonstrate that biotin-labeled oligosaccharides may serve as a diagnostic tool to detect *T. canis* and *T. cati* infections in humans.
